# Decoupling Adsorption and Photocatalysis: Addressing the Dark Adsorption Pitfall in Catalyst Ranking

**DOI:** 10.1021/acsomega.5c09004

**Published:** 2026-01-07

**Authors:** Anna Dougan-Bacha, Jordan E. Cox, Sarah K. St. Angelo

**Affiliations:** Department of Chemistry, 2272 Dickinson College, 28 N. College Street, P.O. Box 1773, Carlisle, Pennsylvania 17013, United States

## Abstract

Heterogeneous photocatalysis relies on the adsorption of the target molecule to the surface of the catalyst and the photocatalytic action of the material to degrade target molecules. In many reports, a 30 min dark adsorption time is used prior to exposure of the system to light and initiation of photocatalysis with no discussion of differences in adsorption rate or capacity when comparing catalysts—when adsorption rates or capacities are very different, misleading catalyst rankings may result. Even closely related materials, such as the ZnO-based photocatalysts discussed here, adsorb target molecules at different rates, have different adsorption capacities, and have apparently different photocatalytic rates. When typical methods for evaluating photocatalysts are used, the adsorption effects are not accounted for. Here, we illustrate potential concerns while evaluating photocatalysts and present a simple, low-concentration method to possibly address—and avoid—adsorption effects. Interestingly, catalyst rankings change when adsorption effects are minimized by circumventing dark adsorption using the low concentration approach presented.

## Graphical abstract

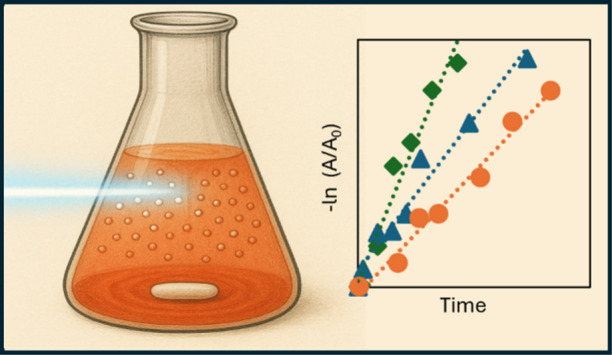

## Introduction

Semiconducting metal oxides, such as ZnO and TiO_2_, are widely used as photocatalytic materials.^1^ With various morphologies and in combination with metal nanoparticles or other semiconductors, there has been much reported work in this area. One application of semiconducting metal oxides and related hybrid nanomaterials is the photocatalytic degradation of environmental pollutants in water, such as dye waste effluent from the textile industry. The photogenerated reactive species on the metal oxides can degrade adsorbed dyes or other pollutants by oxidation or reduction pathways, often mineralizing pollutants.^2^

Among traditional water treatment approaches, photocatalysis stands out as a promising method for degrading diverse toxic and organic pollutants present in wastewater.^2^ Adsorption is used for remediation of dyes and other pollutants in wastewater,^3–7^ and ZnO is an excellent sorptive material.^8,9^ Because the contributions of adsorption to the overall photocatalytic remediation process are not consistently discussed, reported catalytic rates are often actually a convolution of the two processes, leaving room for questions about how adsorption and photocatalysis processes contribute to the reported rate constants and overall remediation of dye target molecules for a given system.^2^

Azo dyes are often used as probe molecules for evaluating photocatalysts. Congo red (Figure 1) is an anionic azo-dye, a class of dyes associated with the textile industry, and it or related dyes are often used as the target molecule for pollutant remediation and photocatalysis studies. Azo dyes, so named because they contain one or more −N=N– azo groups, represent the largest class of dyes with >60% of the world’s dye production as of 2022.^10^ They strongly absorb in the visible region, allowing for simple measurements of concentration with commonly available UV–vis spectrometers. Azo dyes are also indicated as an environmental pollutant in waterways as a result of textile dying and are considered toxic and carcinogenic. Their large molar absorptivities, while useful in the laboratory, block light in aquatic systems, negatively affecting aquatic life. Remediation of azo dyes from aquatic systems by photocatalysis provides motivation and purpose for much of the reported work.

**Figure 1:**
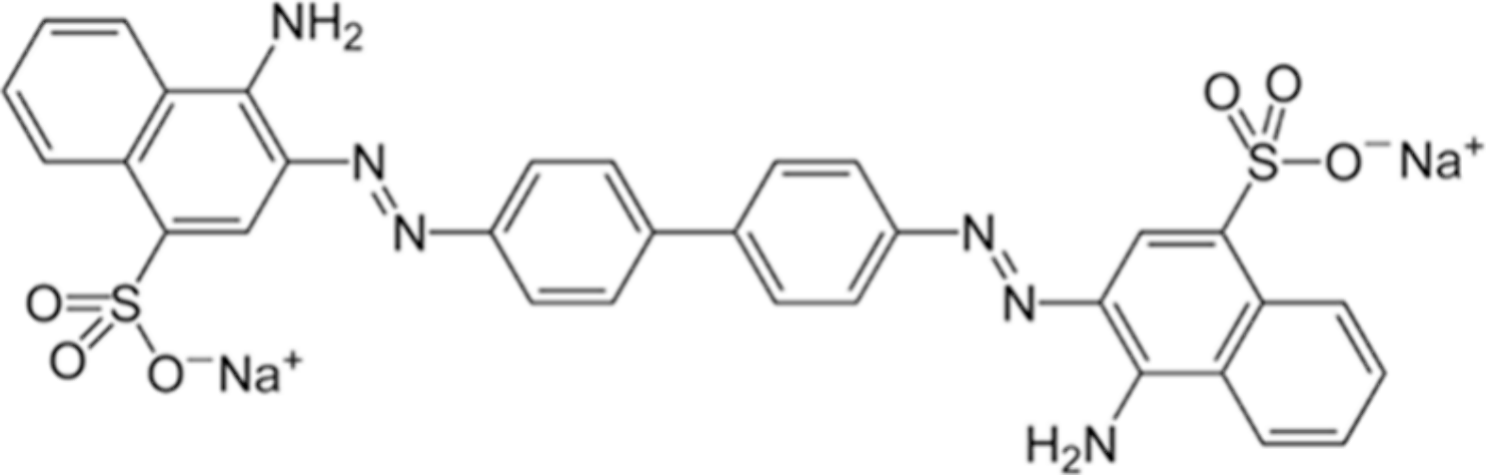
Structure of Congo is shown in red. Congo red is an azo-dye commonly used as a model pollutant.

Reported heterogeneous photocatalysis procedures generally include preparation of reaction solutions of the target molecule (like Congo red) and photocatalyst, a period of dark adsorption to bring target molecules to the photocatalyst surface, and exposure to light to initiate the photocatalytic reaction that degrades the target molecule. To determine the kinetics of degradation, it is common for researchers to periodically extract samples from the reaction solution, remove the photocatalyst by sedimentation or filtration, and measure the optical absorbance of the remaining solution. Over time, the decreasing concentration of the probe molecule allows for calculating a rate constant using pseudo-first-order kinetics, and the decreasing absorbance is generally attributed to the photocatalytic degradation of the probe molecule.

A simplified experimental scheme for photocatalytic degradation of the dye in water is shown in Figure 2. For the photocatalytic degradation to proceed, the target molecules must adsorb to the catalyst surface. After adsorption, the material is illuminated to generate reactive species that interact with the adsorbed pollutant, and degradation products are released. Even though it is typical for a 30 min dark adsorption period to precede photocatalysis,^11–19^ some researchers use longer times,^20–25^ and others either do not use or do not clearly note a dark adsorption period.^26–35^ This may be problematic because materials adsorb target molecules at different rates, and even if adsorption–desorption equilibrium is reached, different but related materials used for comparison do not necessarily have the same equilibrium position.

**Figure 2:**
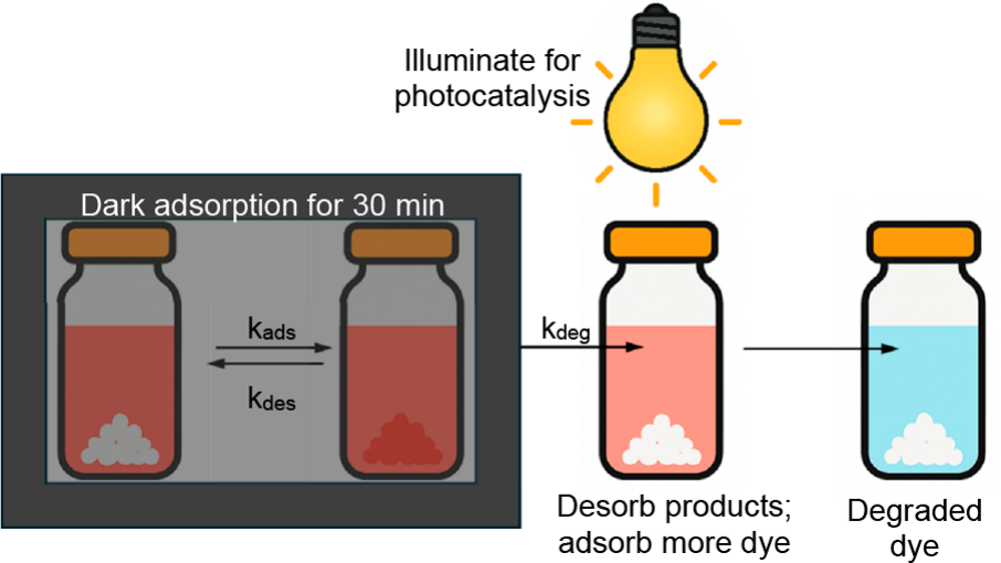
Steps for heterogeneous photocatalytic degradation of the dye from water. Each *k* value represents a rate constant associated with a step in the process. Complete photocatalysis of the dye means that ultimately all dissolved dye is degraded.

The Langmuir–Hinshelwood model is often used to describe heterogeneous photocatalysis and the kinetics and equilibrium that make up heterogeneous photocatalysis.^36^ It is first assumed that equilibrium is established between adsorption and desorption of target molecules to the catalyst surface, as expressed by

1 $$K=\frac{k_{ads}}{k_{des}}$$

where *K* is the equilibrium constant, *k*_*ads*_ is the rate constant for adsorption, and *k*_*des*_ is the rate constant for desorption. The rates of adsorption and desorption can be expressed in terms of the constants and concentration terms of the target molecule, R, in solution and on the surface as

2 $$v_{ads}=k_{ads}\left(1-\theta \right)\left[R\right]$$

3 $$v_{des}=k_{des}\theta$$

where *v*_*ads*_ and *v*_*des*_ are rates of adsorption and desorption, respectively, θ is the fractional surface coverage, and [R] is the concentration of the target molecule in solution. When equilibrium is reached and the rates of adsorption and desorption are equivalent, the above equations can be combined to yield a value for the fractional surface coverage as

4 $$\theta =\frac{K\left[R\right]}{1+K\left[R\right]}$$

The rate of degradation, *v*_*deg*_, can be expressed in terms of a change in concentration with time as

5 $$v_{\deg}=-\frac{d\left[R\right]}{dt}$$

which is then used as an integrated first-order approach to obtain rate constants as

6 $$\ln\left[R\right]=-k_{\deg}t+\ln{\left[R\right]}_{0}$$

where *k*_*deg*_ is the rate constant for degradation, *t* is time, and [R]_0_ is the initial concentration of the target molecule. Photocatalytic degradation can occur only for molecules adsorbed to the catalyst surface, so the rate of degradation can also be expressed as

7 $$v_{\deg}=k_{\deg}\theta$$

Practically speaking, concentrations of target molecule in solution, [R], are more easily measured than values of *θ.* When eqs 4, 5, and 7 are combined, the resulting expression is the Langmuir–Hinshelwood kinetic model:

8 $$-\frac{d\left[R\right]}{dt}=k_{d}\frac{K\left[R\right]}{1+K\left[R\right]}$$

If equilibrium is not reached or the equilibrium positions for test catalysts are not equivalent, then differences in rates of overall degradation may be strongly affected by the adsorption–desorption equilibrium. While the Langmuir–Hinshelwood model can be used to describe the kinetics of photocatalysis, many reports rely on a pseudo-first-order kinetics treatment of data by using changing concentrations of target molecule in solution.

## Photocatalytic Degradation

Semiconducting metal oxides, such as ZnO, can convert sufficiently energetic photons to highly reactive species that may then find use in other processes, like catalysis of organic reactions, photovoltaics, water splitting for hydrogen production, CO_2_-reduction, or pollutant remediation.^37,38^

Bulk ZnO has a direct band gap of 3.37 eV, or energy equivalent to 368 nm UV light. When exposed to light (*hν*) in the near UV region, electron (*e* ^*–*^) hole (*h* ^*+*^) pairs, also known as excitons, are generated:

$$\mathrm{ZnO}+h\nu \to \mathrm{ZnO}\left(e^{-},h^{+}\right)$$

If oxygen and water are accessible, then the pair may interact with the molecular species to form highly reactive radical species:

$$\begin{array}{l} e^{-}+O_{2}\to {O_{2}}^{\cdot -}\\ h^{+}+{\mathrm{OH}}^{-}\to {\mathrm{OH}}^{\cdot } \end{array}$$

Superoxide radical (O_2_^•–^) and hydroxyl radical (OH^•^) are available to react with other species to drive reduction and oxidation pathways, respectively.^13^ The bandgap, and therefore minimum photon energy needed to surpass it, may be altered by doping, size effects, and coupling to other materials; however, recombination of the electron–hole pair and resultant deactivation of the reactive species can hinder subsequent reactions of interest.

In order for photocatalysis to proceed, target molecules must be adsorbed onto the surface of the photocatalytic material. By nature, surface adsorption brings the target molecule from solution and confines it to a solid surface. Surface adsorption, however, does not mean that photocatalytic degradation of the target molecule has occurred. The target can be removed from the solution but not degraded. By evaluating the concentration of target molecule in solution, researchers measure how much target molecule has been removed from solution *by any means*. In other words, removal by adsorption and removal by adsorption plus photocatalysis look the same when the concentration of the target molecule is decreased. Additionally, the time allotted for dark adsorption affects the initial concentration of the target molecule in solution when photocatalysis is initiated. Differences in adsorption rates or equilibria affect the concentration of target molecules in solution at the start of photocatalysis and affect the initial rates measured.

## Practical Difficulties in Evaluating Photocatalytic Materials

When evaluating related materials, comparing their overall photocatalytic performance is confounded by these competing processes. In an extreme example, a material rapidly adsorbing the target molecule could appear to be the most effective photocatalyst if the only measure is the target molecule remaining in solution.

We have noted that solids removed from suspension at various time points have distinctly different loadings of target dye based on their color. Related ZnO or ZnO-Ag materials at the same material concentration of 1.6 mg/mL and Congo red concentration of 4 ppm show different adsorption of the dye after the dark adsorption period and sedimentation by centrifuge (Figure 3a). In what would be an initial time point measurement after dark adsorption, two of the materials (labeled 1 and 3) have visibly removed most of the dye present, as indicated by a nearly colorless supernatant. The other materials (labeled 2 and 4) are also sedimented but have notable red dye remaining in the supernatant.

**Figure 3:**
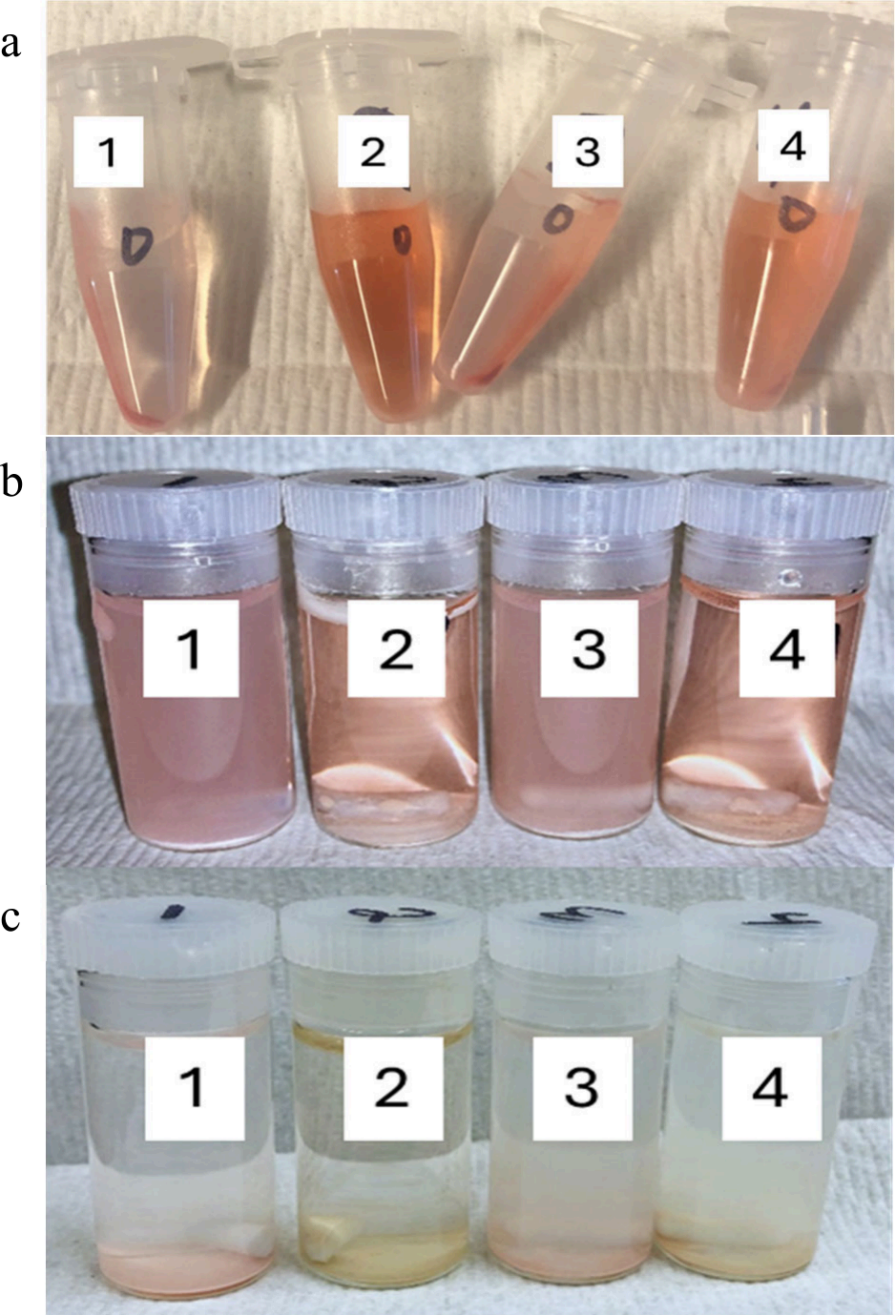
ZnO and ZnO-Ag materials and dye adsorption and photocatalytic degradation. (a) After 30 min dark adsorption with 4 ppm Congo red in water followed by sedimentation, dye is mostly adsorbed to solids in samples 1 and 3, leaving a colorless supernatant. Samples 2 and 4 still show dye in the supernatant, indicating less surface adsorption. (b) The same materials exposed to Congo red at 4 ppm before exposure to 365 nm light. Similar colors are noted. (c) The samples in (b) after exposure to 365 nm light for 40 min and subsequent gravity sedimentation. The solids at the bottom of the vials show different extents of photocatalytic degradation of the dye.

Further, when light exposure initiates photocatalytic break down of Congo red, different colors of the solids indicate differing photocatalytic rates. In Figure 3b, the materials are shown after the 30 min dark adsorption period. The colors are similar because no degradation of the dye has yet occurred, but the clarity of the solutions is different, likely due to differences in adsorbed versus free dye in solution. In Figure 3c, the same samples are shown after 40 min of exposure to 365 nm light. The solids were allowed to settle under gravity. Each supernatant is nearly clear and colorless, but the solids in samples 1 and 3 are pinkish, indicating incomplete degradation of the Congo red dye. If only relying on the absence of dye absorbance in the supernatant, all samples would be judged to have degraded the dye within the measurement period.

In a typical photocatalysis experiment using related ZnO-based materials performed by periodically sampling the reaction mixture, sedimenting solids, and measuring absorbance values of the target molecule, first-order kinetics plots can be obtained, such as those show in Figure 4a. Rate constants of (4.6 ± 0.1) × 10^–2^, (3.5 ± 0.3) × 10^–2^, and (1.4 ± 0.4) × 10^–2^ min^–1^ were obtained. In ranking compounds, we found compound *k*(A) > *k*(C) > *k*(B) in terms of apparent rate constant; however, the compound with the smallest rate constant started with the lowest apparent absorption of dye in the supernatant after the 30 min dark adsorption period, as seen in Figure 4b. Again, because the solids were sedimented with adsorbed dye and the dye absorption of the supernatant was measured, it is not possible to distinguish whether the dye was degraded by photocatalytic processes or only physically removed.

**Figure 4:**
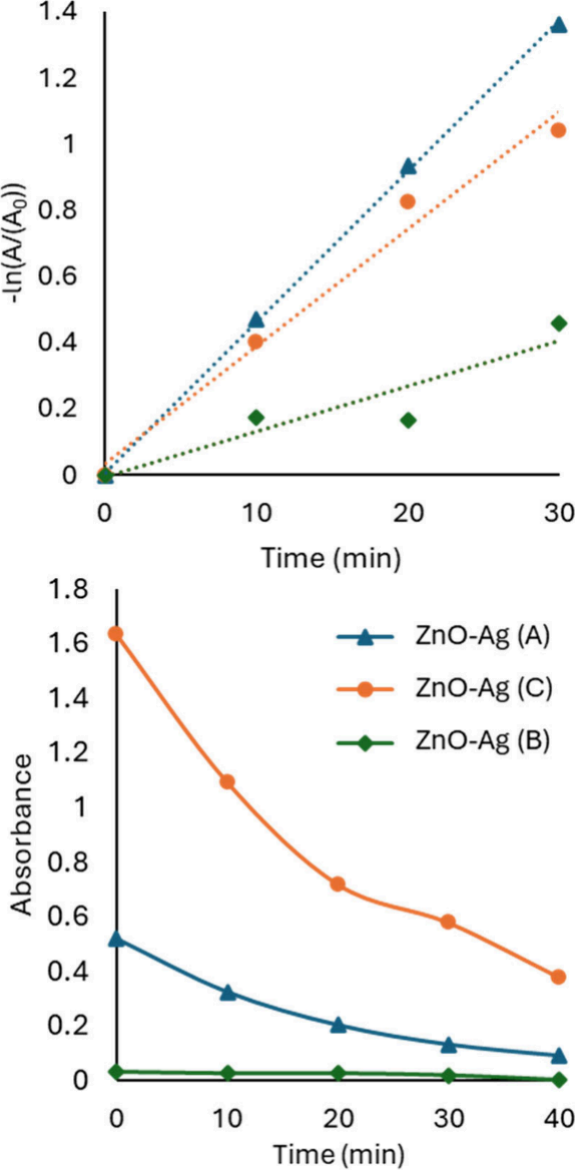
Kinetics of photocatalysis for three related ZnO-Ag compounds with 30 min of dark adsorption. (a) The analysis by first-order kinetics seems to indicate a larger rate constant for degradation for the compound with the steepest slope. Rate constants are (4.6 ± 0.1) × 10^–2^, (3.5 ± 0.3) × 10^–2^, and (1.4 ± 0.4) × 10^–2^ min^–1^. (b) The absorbance of the target molecule over time shows three very different starting concentrations for *t* = 0 (after dark adsorption). Most of the target molecule was removed from the surrounding media via adsorption of the compound with the smallest apparent degradation rate constant.

## Rates of Adsorption for Related Materials

We can measure the decreasing concentration of supernatant over time due to adsorption only. For example, three related ZnO-Ag hybrid materials were synthesized, heat treated at the same temperature (200 °C), and prepared at the same concentration (1.7 mg/mL) with the same concentration of target molecule (40 ppm Congo red dye). Solutions were shielded from light with continuous stirring. Aliquots were removed at time intervals, and the solids were sedimented by centrifugation. The supernatant absorbance values were measured at the maximum absorbance of the dye to record its decreasing concentration over time as shown in Figure 5a. Various rates of adsorption were observed within the dark adsorption period, even for closely related compounds, and the data were plotted with pseudo-first-order kinetics in Figure 5b. As shown in Figure 5b, there is as much as nearly a 10-times difference in rate constant for removal of dye via adsorption only: the slopes are 0.155 ± 0.008, 0.104 ± 0.003, and 0.016 ± 0.003 min^–1^. Because the same materials and conditions were used in Figure 4 and Figure 5, we can directly compare the rate constants for the materials. The “slowest” material, ZnO-Ag (B), in the typical photocatalysis experiment in Figure 4 is notably “faster” in Figure 5 due to the effects of the rate of adsorption.

**Figure 5:**
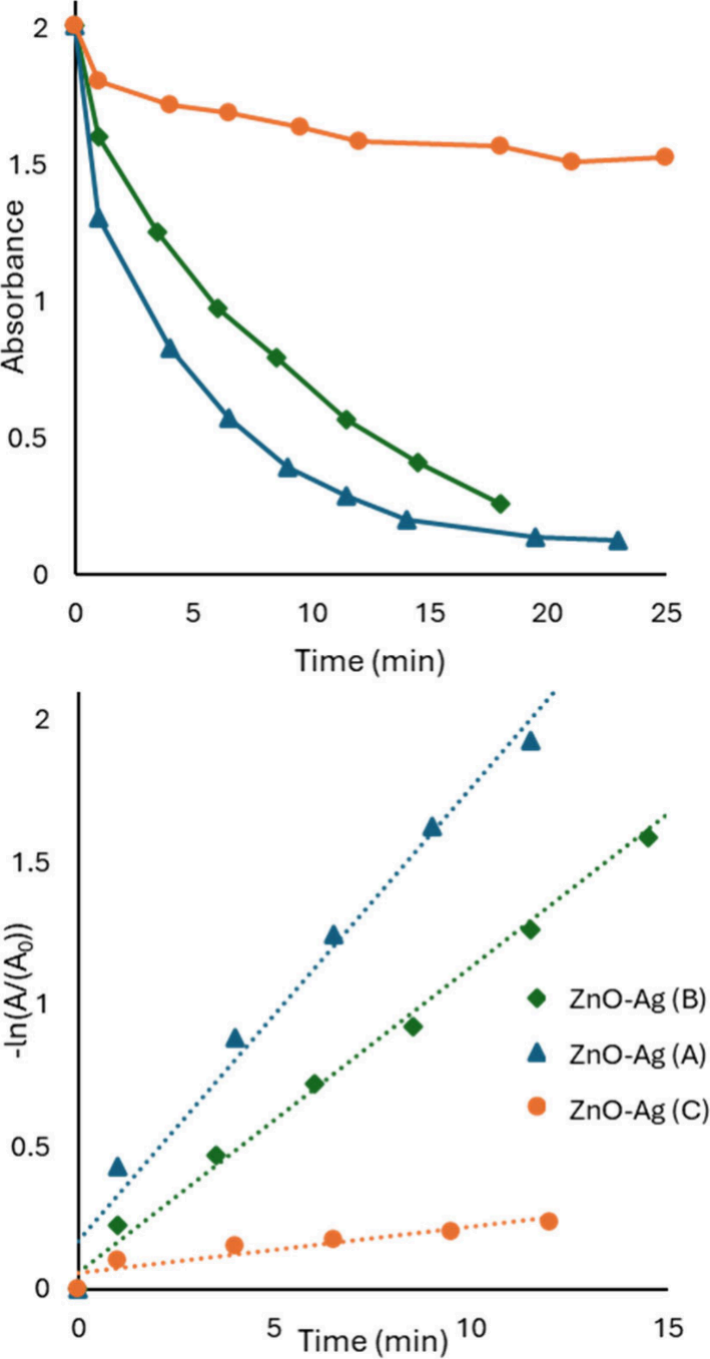
Kinetics of dark adsorption for related ZnO-Ag solids adsorbing Congo red dye. (a) Decreasing absorbance of Congo red in the supernatant over time. (b) Pseudo-first-order treatment of the data in (a). The related compounds adsorb Congo red at different rates within the dark adsorption period.

## Adsorption Capacity at Equilibrium

For a series of related ZnO-Au hybrid materials, the material adsorption capacities for a variety of Congo red dye concentrations are very different. The same reagents were used to prepare the hybrid materials except for the type of Au nanoparticles added during synthesis. For example, when ZnO was precipitated with as-synthesized quasi-spherical particles, rod-shaped particles, and platelet type particles to investigate photocatalytic effects based on particle type, different adsorption capacities were observed at various Congo red concentrations. Synthesis of the anisotropic particles and spheres used as seed particles requires soft templating with micelles created with strong concentrations of surfactants, cetyltrimethylammonium bromide/chloride (CTAB/C).^39–44^ The surfactants coat the Au NPs and may cover the overall hybrid materials. When we investigated the remediation of Congo red dye with ZnO and anisotropic AuNPs, we found an increased adsorption capacity. This is not surprising given that CTAB-modification of materials can be used to enhance adsorption of Congo red.^45,46^ The same amounts of ZnO-Au material were suspended in varying concentrations of Congo red dye, and the mixtures were allowed to interact with stirring in the dark for 24 h to reach equilibrium. Dye concentrations used here were much higher than those used in photocatalysis. The solids were isolated, dried, and spectroscopically evaluated with a diffuse reflectance with an integrating sphere UV–vis spectrometer. Interestingly, the adsorption capacities were not consistent among these related materials, as shown in Figure 6. The amount of dye adsorbed to the solid affects the maximum absorbance in diffuse reflectance, as it would in transmission spectroscopy.

**Figure 6:**
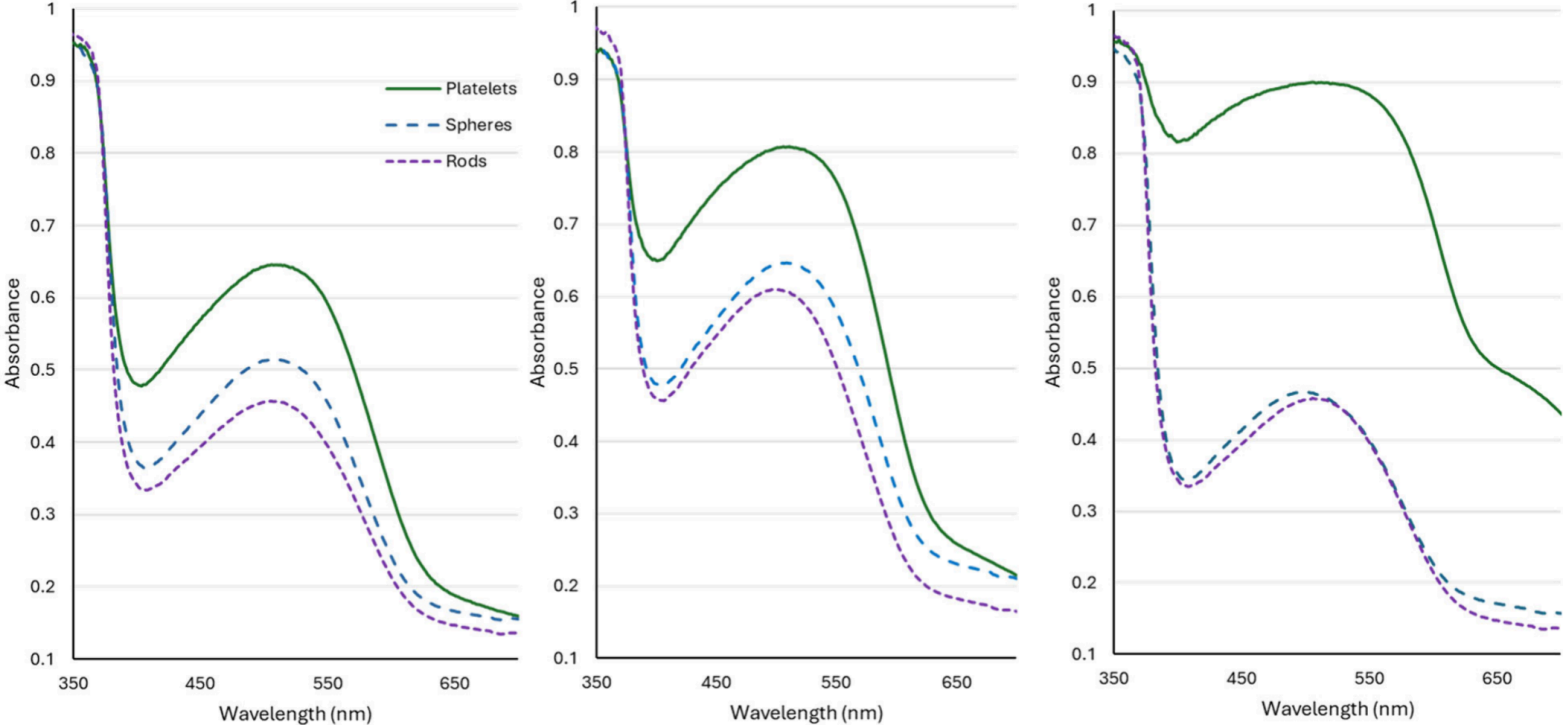
Equilibrium adsorption of Congo red at various concentrations to ZnO-Au hybrid nanomaterials. Each plot shows the adsorption of ZnO-Au hybrid materials made with spherical, rod-like, and platelet shaped Au. (a) Adsorption of 10 ppm, (b) 40 ppm, and (c) 200 ppm Congo red dye.

For each of the dye concentrations shown in Figure 6 (10, 40, and 200 ppm), the ZnO-Au sample made with platelets captures the most dye from solution. This is likely due to increased surfactant used in the synthesis of the platelets compared to the other Au particle types; however, when comparing these materials after a 30 min dark adsorption, we were unable to initiate photocatalytic degradation measurements because so little dye remained in solution at the initial time point.

Depending on the use of a dark adsorption period, its length, and the concentration of target molecule, the starting point for the amount of adsorbed target molecule and, therefore, the concentration of target molecule in solution can be very different. The dark adsorption period, regardless of length, removes target molecules from the solution but does not apparently degrade them by photocatalysis or other means. If degradation is to be monitored, adsorbed and free target molecules should be included in the measured concentration that informs a discussion of the kinetics of degradation. Typical methods discussed previously include centrifugation or filtration steps to remove heterogeneous particles before determining the optical absorbance of the target molecule remaining in solution. Heterogeneous catalysts at concentrations typically used (e.g., 1 mg/mL) create turbid suspensions that scatter light and obscure the absorbance of the target molecule. Further, at these catalyst concentrations material flocculates, so reaction mixtures are typically stirred to prolong suspension and aid mixing.

## Low Concentration Method to Evaluate Photocatalytic Degradation without Adsorption Effects

At sufficiently low particle concentrations, scattering by suspended solids does not preclude using transmission spectroscopy to measure the absorbance of target molecules, like Congo red dye. For example, the absorbance (more accurately, the scattering and scattering plus absorbance or extinction) over time of a suspension of ZnO-Ag and ZnO-Ag plus Congo red is shown in Figure 7. There is a small decrease in extinction noted for the ZnO-Ag plus Congo red line that is not present in the ZnO-Ag only line. This decrease occurs mostly within the first minute and may represent slight degradation of Congo red, perhaps due to a low concentration of reactive surface species on the ZnO-Ag when exposed to ambient light.

**Figure 7:**
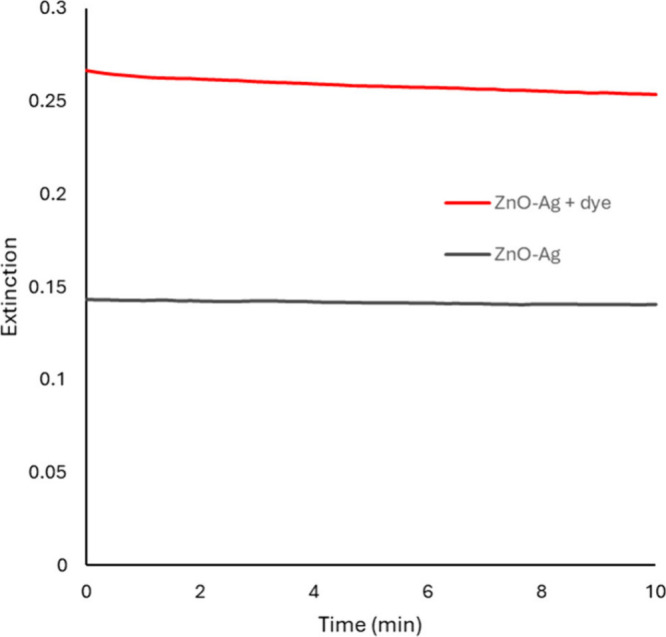
Extinction over time for suspended ZnO-Ag (black) and ZnO-Ag with Congo red (red). The suspensions produce stable extinction values over at least 10 min, even without stirring.

ZnO-Ag particles were suspended in solution (42 μg/mL) with and without 1 ppm Congo red. The suspensions were mixed directly in a transmission cuvette, and extinction values were collected at 498 nm, the absorption maximum for Congo red. Suspensions were not stirred during data collection. 498 nm light is not sufficiently energetic to initiate the formation of reactive species in ZnO and is lower energy than the plasmon maximum for Ag nanoparticles (∼400 nm). The lower trace (black) shows the stability of the particle suspension, and the upper trace (red) shows the stability of the particle suspension with Congo red dye.

Because the extinction values were sufficiently stable over time and a difference in extinction due to absorption of Congo red dye could be measured even at low concentrations, we were able to perform photocatalysis without dark adsorption and without sedimenting solids. Avoiding dark adsorption and sedimentation allows us to measure the overall process without dye losses due to adsorption without photocatalysis. Example results of kinetics analysis and associated decreasing concentrations when dark adsorption is avoided are shown in Figure 8.

**Figure 8:**
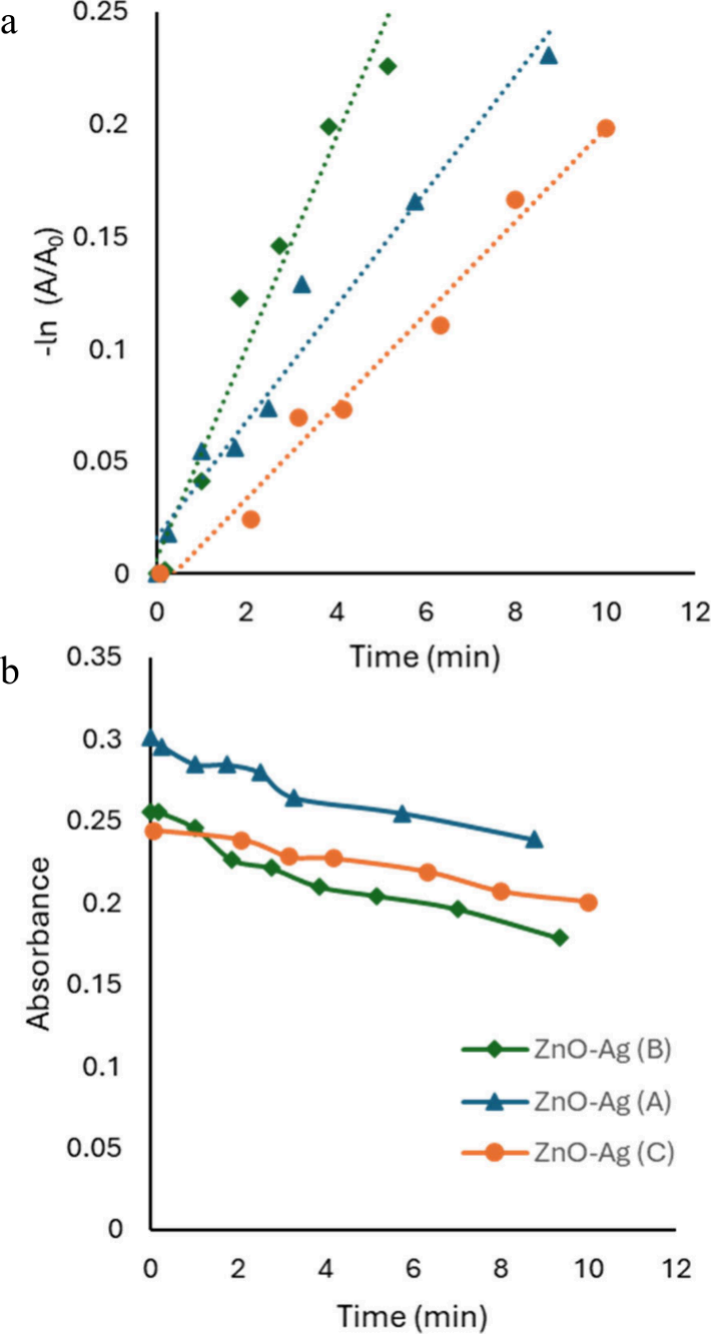
Kinetics of photocatalysis for ZnO-Ag materials without dark adsorption and sedimentation. (a) Pseudo-first-order treatment of the data. The order of photocatalytic rate constants changes when dark adsorption and sedimentation steps are avoided. (b) Shows the decrease in absorbance over time of the suspended solids and dye. The decreasing absorption is a result of degradation of dye.

The rate constants for photocatalysis were again taken from slopes of pseudo-first-order kinetics plots. Figure 8a yields rate constants of (4.70 ± 0.04) × 10^–2^, (2.57 ± 0.02) × 10^–2^, and (2.06 ± 0.12) × 10^–2^ min^–1^. The same materials used in Figure 8 were used in Figure 4 and 5; however, the apparent ranking of photocatalytic remediation of the probe molecule is again different. The effects of adsorption are reflected here, where adsorption and photocatalysis begin at the same initial time point. Interestingly, the material ZnO-Ag (C) is distinctly slower as a sorbent material by a factor of ∼7–10 but is comparable to the other materials when photocatalysis and adsorption are not separated (factor of ∼1.2–2.3). At low concentrations and without dark adsorption, the observed degradation rate better corresponds to the actual photocatalysis efficiency of each sample.

In comparing the rate constants for the removal of dye, it is clear that the method affects the ranking of materials. Even with the same materials, adsorption effects can dominate the apparent rate constants. Values for materials discussed here are summarized in Table 1.

**Table 1:** Apparent Rate Constants (*k*) for Dye Degradation/Removal for ZnO-Ag Materials

Dye Removal Parameters	ZnO-Ag (A) (min^–1^)	ZnO-Ag (B) (min^–1^)	ZnO-Ag (C) (min^–1^)
Photocatalysis with dark adsorption and sedimentation	(4.6 ± 0.1) × 10^–2^	(1.4 ± 0.4) × 10^–2^	(3.5 ± 0.3) × 10^–2^
Adsorption only	0.155 ± 0.008	0.104 ± 0.003	0.016 ± 0.003
Photocatalysis without dark adsorption and sedimentation	(2.57 ± 0.02) × 10^–2^	(4.70 ± 0.04) × 10^–2^	(2.06 ± 0.12) × 10^–2^

When evaluating removal of dye by typical dark adsorption, photocatalysis, and removal of solids by sedimentation, the ranking of catalyst materials in our system is *k*(A) > *k*(C) > *k*(B). When adsorption of dye is considered without photocatalysis, the rank ordering changes, and one material emerges as having about 10× slower adsorption rate: *k*(A) > *k*(B) ≫ *k*(C). When dark adsorption is avoided by using lower concentrations of suspended catalyst and dye such that visible extinction can be measured directly, the ordering changes again as *k*(B) > *k*(A) > *k*(C). The dark adsorption period can effectively obscure photocatalytic rates, even among closely related compounds, when there are meaningful differences in rates of adsorption by the materials.

## Conclusions

There are numerous factors to consider when investigating photocatalytic materials for degradation of target molecules in solution. These include the amount of photocatalyst, concentration of target molecule, temperature, pH of surrounding media, length of dark adsorption period, wavelength of photocatalytic excitation, and power of excitation, and all can affect the observed rates (and rate constants) of apparent degradation of the target molecule. Cautionary reports of how to better design experiments, measure photocatalysis, and analyze results are important background for those undertaking such experimental work.^36,47–53^ While the approach presented here does not address all possible concerns, it does offer opportunities to gain insight regarding how much dark adsorption may affect perceived photocatalytic rates when using a common experimental design for such work. Dark adsorption, when not specifically considered, may lead to problematic assumptions about the apparent degradation of target molecules. By avoiding dark adsorption and using lower concentrations of catalyst and target molecules, the overall effect can be observed. When comparing the efficacy of related materials in a self-consistent system, researchers may be able to avoid misattributing decreasing concentrations of target molecules to photocatalytic degradation when adsorption is a stronger factor. Specifically, our results demonstrate that ignoring differential dark adsorption can lead to an overestimation of apparent photocatalytic rate constants by up to 1 order of magnitude and can even invert the ranking of catalyst performance in self-consistent systems.

## Supplementary Material



## Notes

TEM/EDS as well as Fourier transform analysis for the ZnO-Ag materials (PDF)

## †

Department of Chemistry, Columbia University, New York, New York 10027, United States
