# Supplementary material for: Decoupling Adsorption and Photocatalysis: Addressing the Dark Adsorption Pitfall in Catalyst Ranking
Source: ACS Omega. 2026 Jan 7;11(2):2289–96. doi: 10.1021/acsomega.5c09004 (PMC12824761; doi:10.1021/acsomega.5c09004)
Supplement: Supplementary file 1 [file ao5c09004_si_001.pdf]

Supporting Information

Decoupling Adsorption and Photocatalysis: Addressing the Dark Adsorption Pitfall in Catalyst Ranking

Anna Dougan-Bacha, Jordan E. Cox,[†] *Sarah K. St. Angelo**

Department of Chemistry, Dickinson College, 28 N. College Street, P.O. Box 1773, Carlisle, PA 17013

* Department of Chemistry, Dickinson College, Carlisle, Pennsylvania 17013, United States; Email: stangels@dickinson.edu

† Present address: Department of Chemistry, Columbia University, New York, New York 10027, United States

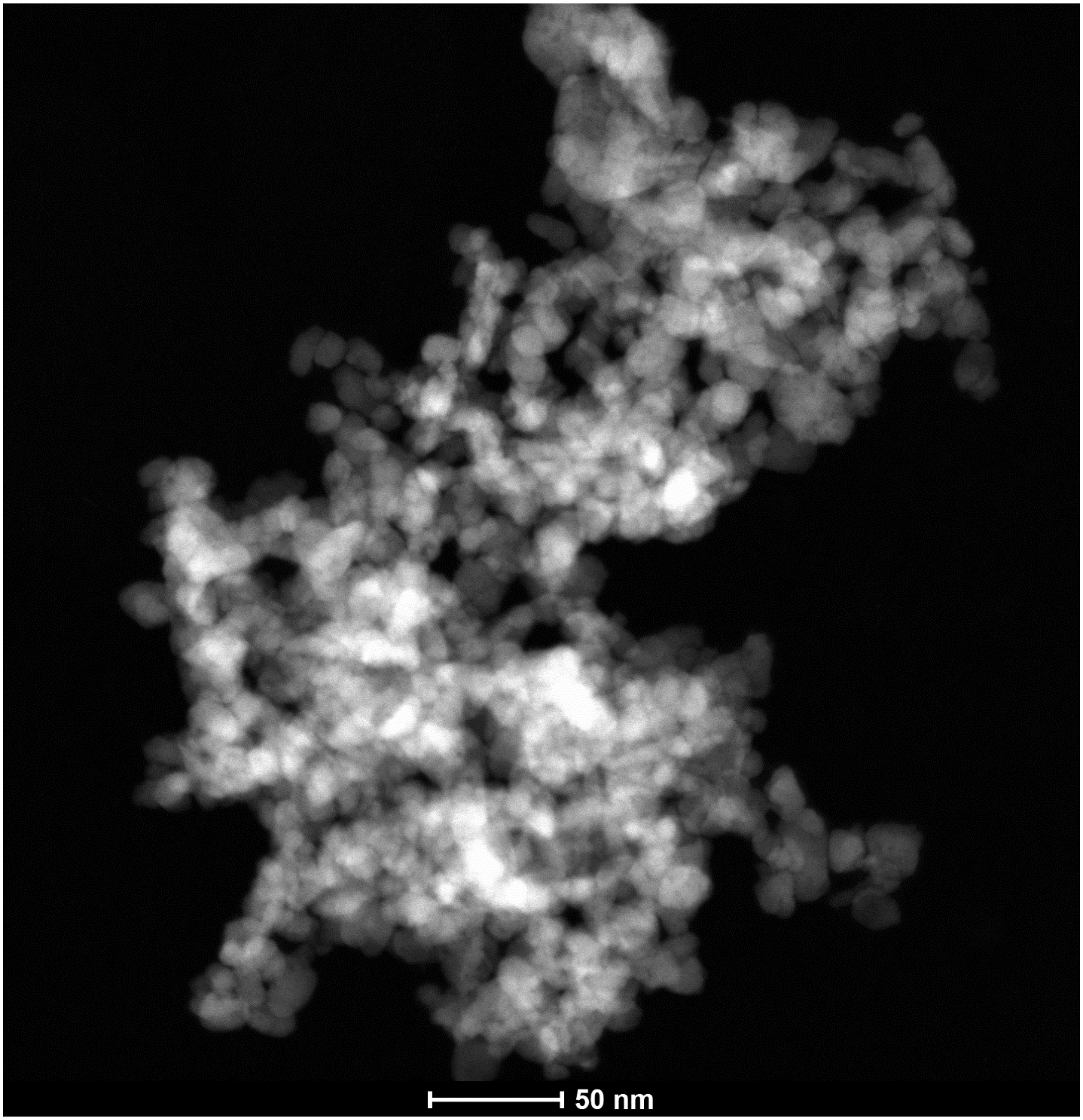

Figure S1: Transmission electron micrograph of ZnO-Ag (A). Most of the sample is ZnO. Ag nanoparticle loading is very low, so broad EDS was inconclusive for presence of Ag.

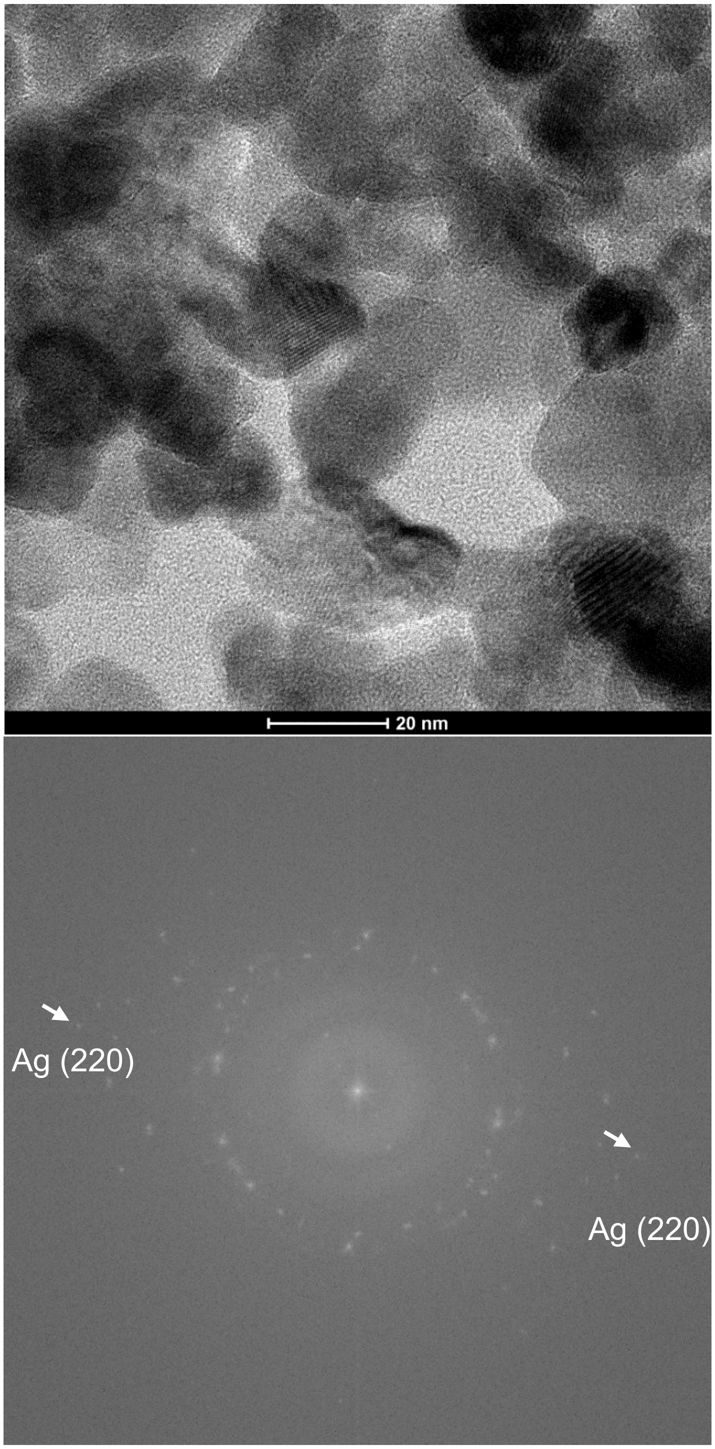

Figure S2: High resolution transmission electron micrograph of ZnO-Ag (A) with two-dimensional Fourier transform (FT). Most spots correlate to lattice spacing associated with ZnO (wurtzite). Spots associated with Ag (220) at 1.44 Å are noted in the FT. Arrows indicate the spots in frequency space associated with Ag lattice spacing. Examples of Ag (200) at 2.04 Å were identified in other similar micrographs.

2025-08-15 B12 15.59.28

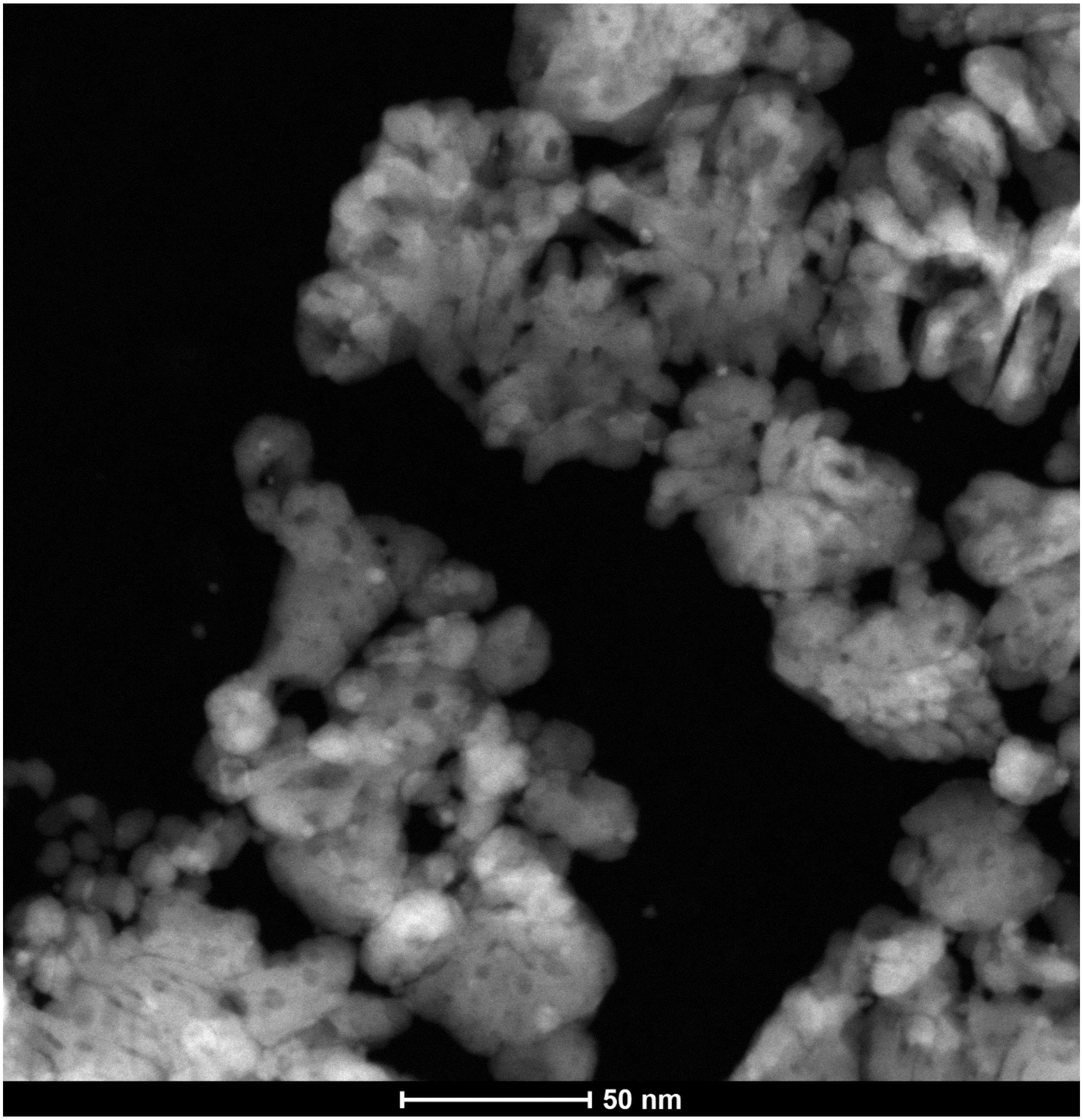

Figure S3: TEM image of ZnO-Ag (B).

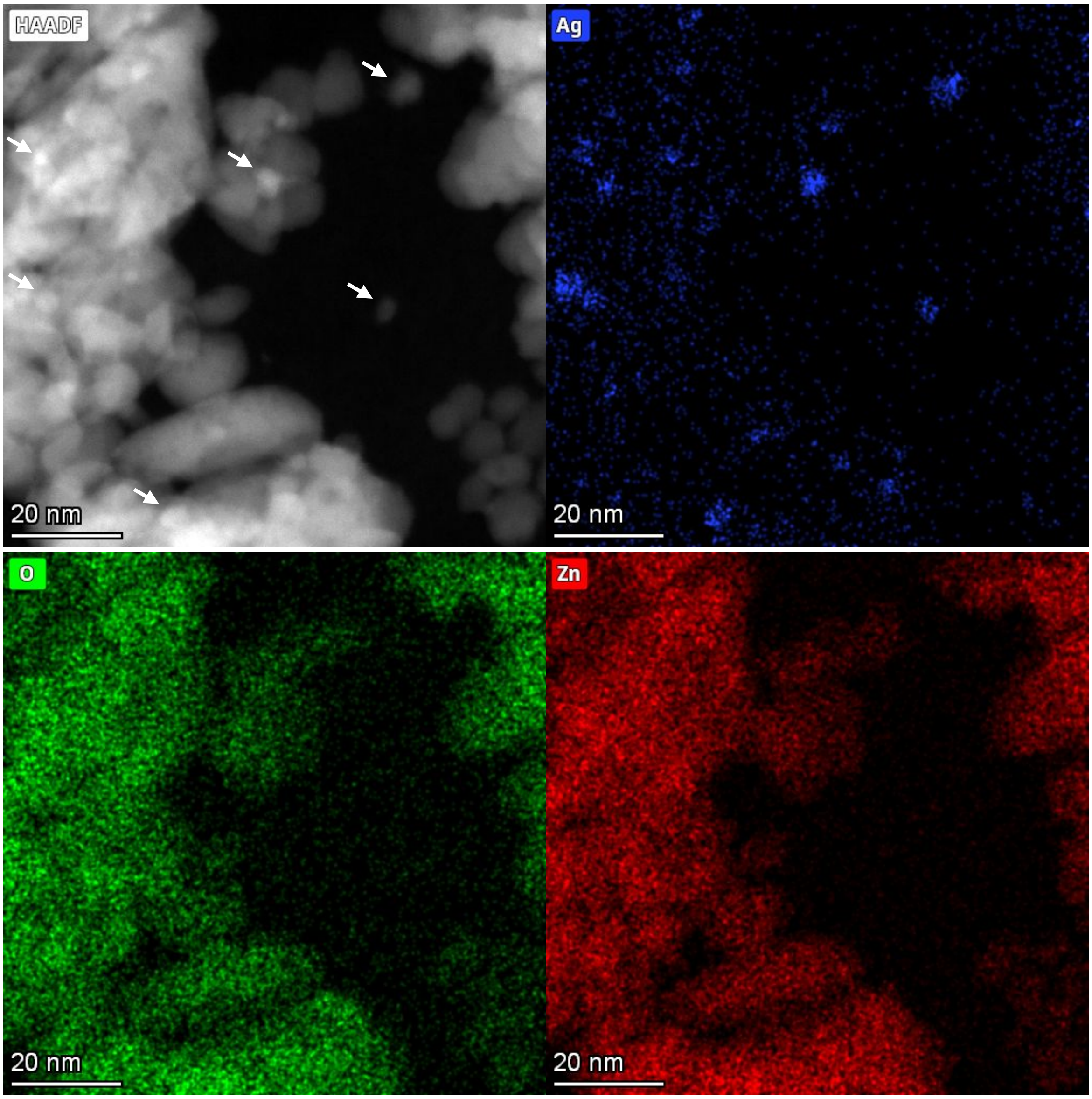

Figure S4: TEM image of ZnO-Ag (B) with associated EDS maps showing elemental mapping for Ag, O, and Zn. Most of the field is represented by ZnO. Several Ag particles indicated with arrows are shown in/on ZnO.

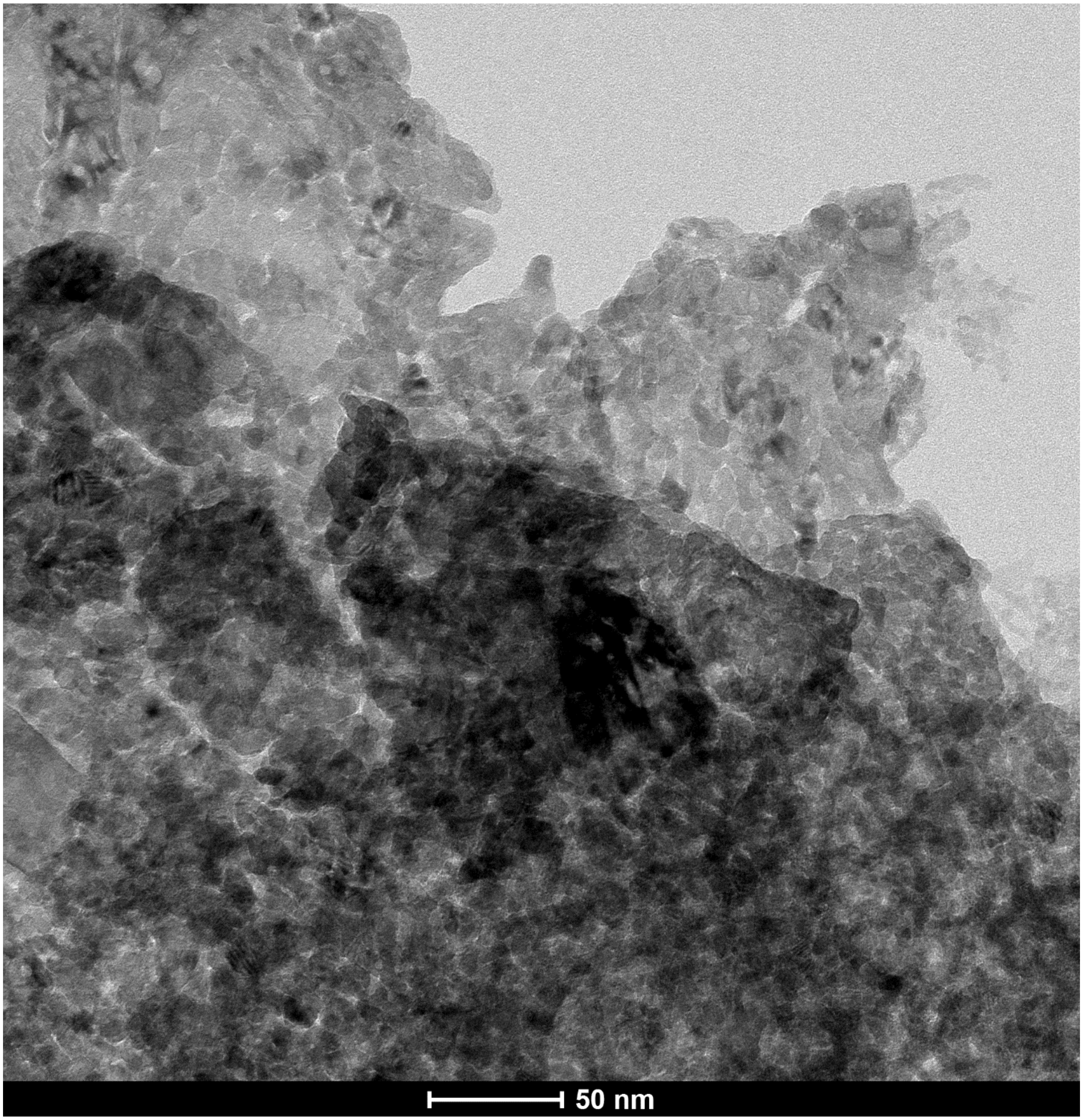

Figure S5: TEM image of ZnO-Ag (C).

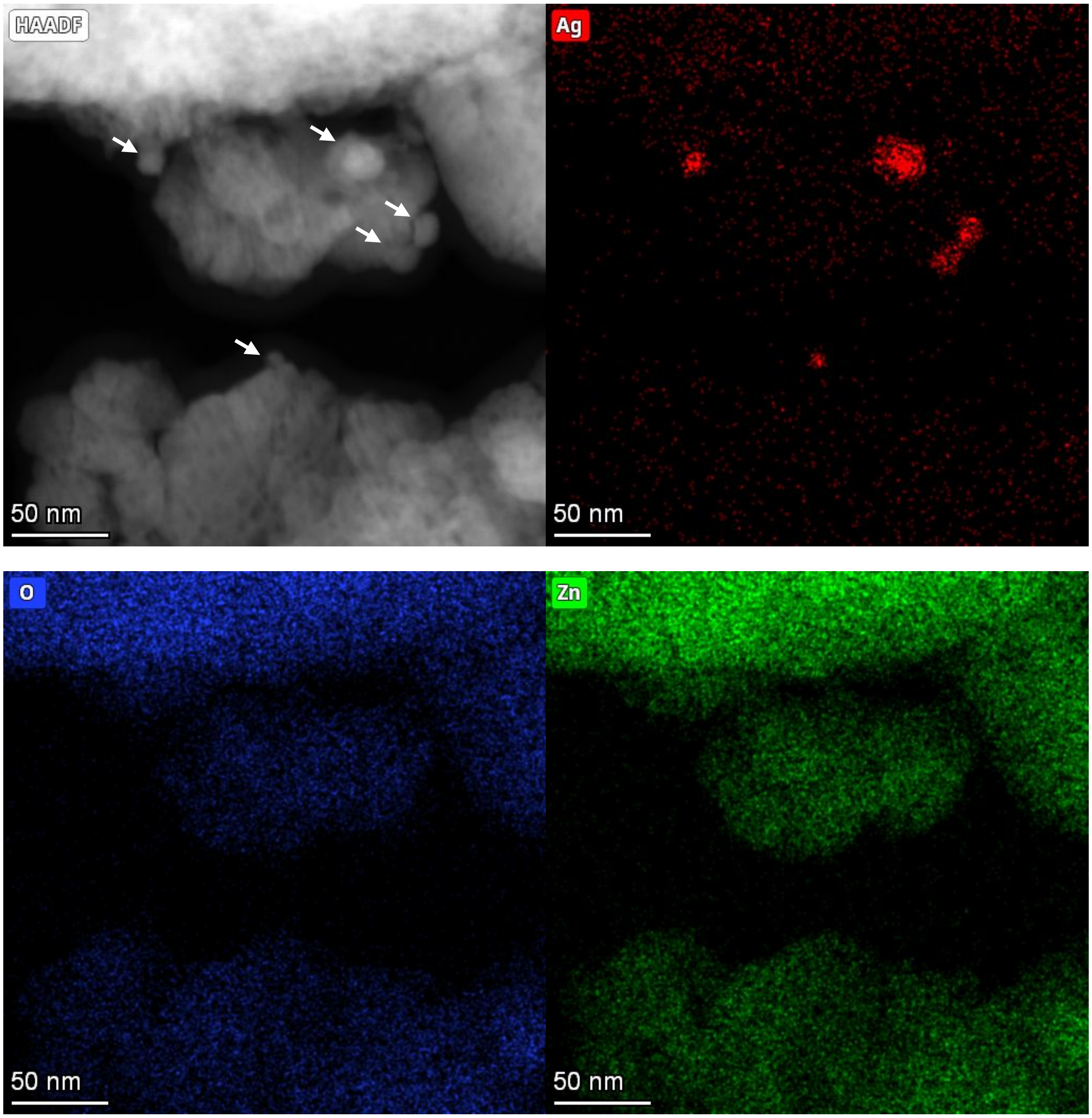

Figure S6: TEM image of ZnO-Ag (C) with associated EDS maps showing elemental mapping for Ag, O, and Zn. Most of the field is represented by ZnO. Several Ag particles indicated with arrows are shown in/on ZnO.
